# Presence of micro-mesoplastics in beaches and sediments of São Francisco do Sul (Brazil)

**DOI:** 10.1007/s10661-026-15277-2

**Published:** 2026-04-24

**Authors:** Elisângela S. Lopes-Ricardo, Amarildo O. Martins, Uberson B. Rossa, Eduardo A. W. Ribeiro, André R. Prado, Erik N. Gomes, Claudemir M. Radetski

**Affiliations:** 1 Instituto Federal Catarinense (IFC), Campus Araquari, Curso de Mestrado Profissional Em Tecnologia E Ambiente, Rodovia BR 280, Km 27, Araquari, SC 89245-000 Brazil; 2 Departamento de Química, Universidade Federal de Santa Catarina (UFSC), Rua Rua João Pessoa, 2750 , Blumenau, SC 89036-256 Brazil; 3 Freitag Laboratórios Ltda, Rua Hermann Berndt, 505, Timbó, SC 89120-000 Brazil; 4 Universidade Do Vale Do Itajaí (UNIVALI), Programa de Pós-Graduação Em Ciência E Tecnologia Ambiental, Itajaí, SC 88302-202 Brazil

**Keywords:** Microplastics, Mesoplastics, Anthropogenic beach pollution, Brazilian beaches, Coastal management

## Abstract

The coastal environment is a critical interface for monitoring plastic pollution. This study investigated microplastics (MPs, < 5 mm) and mesoplastics (MePs, 5–25 mm) in Praia Grande, São Francisco do Sul (SC State, Brazil), using stereomicroscopy and ATR-FTIR spectroscopy. Two sites with contrasting urbanization and tourism levels were assessed. Area #1, the more urbanized site, exhibited higher concentrations of MPs (70.44 particles·m⁻^2^) and MePs (25.11 particles·m⁻^2^) than Area #2 (19.11 MPs·m⁻^2^; 6.67 MePs·m⁻^2^). Morphological analysis revealed predominance of fragments in Area #1 and fibers (MPs) or foam (MePs) in Area #2. White, blue, and translucent particles were most abundant, while polymer identification highlighted the dominance of polyethylene, polypropylene, and styrene-butadiene copolymer, reflecting both global production patterns and local anthropogenic inputs. Sediment granulometry and hydrodynamics emerged as primary drivers of plastic retention, modulated by urbanization and tourism. Positive correlations between MePs and MPs indicate that MePs act as transitional reservoirs, contributing to secondary microplastic formation. These findings emphasize the hierarchical interplay of physical and anthropogenic factors in shaping coastal plastic distribution and provide a process-based framework to inform monitoring and management strategies.

**Supplementary Information:**

The online version contains supplementary material available at 10.1007/s10661-026-15277-2.

## Introduction

Plastic pollution is an increasing global challenge for the sustainability and balance of ecosystems, with documented impacts on both the environment and human health (Cholewinski et al., 2022; Deus et al., 2024; Jung et al., 2022; Tian et al., 2023). Recent studies have shown that the accumulation of plastic waste on beaches not only affects visual appeal but also impacts local economies by reducing tourism revenue and increasing the costs associated with beach cleanup efforts (Beaumont et al., 2019; Escrobot et al., 2024; Gago et al., 2020; Hayati et al., 2020; Krelling et al., 2017; UNEP, 2023). The growing presence of microplastics and mesoplastics (M(e)Ps) in ecosystems, largely due to poor waste management and the long persistence of plastics in the environment, has raised serious concerns leading UNEP to disseminate a range of information about this current environmental problem (https://www.unep.org/plastic-pollution).

The coastline, representing the interface between land and sea, is a critical zone for monitoring plastic residues (Abidli et al., 2018; Amparo et al., 2023; Browne et al., 2010; Cavalcante et al., 2020; GESAMP, 2019). It was reported increasing concentrations of microplastics in beach sediments, even in remote areas, underscoring the global reach of anthropogenic impacts (Lloyd-Jones et al., 2023; Niu et al., 2024).

The frequent occurrence of M(e)Ps on sandy beaches highlights the urgent need to identify their sources to support the development of effective mitigation strategies (UNEP, 2021, 2023). However, determining the origin of M(e)Ps in coastal environments is complex due to the diverse and heterogeneous factors contributing to their accumulation (An et al., 2024). These pollutants can be originated from both land-based and marine sources, with common transport pathways including river discharge, stormwater runoff, and improper waste disposal. M(e)Ps, whether primary (manufactured as small particles) or secondary (resulting from the degradation of larger plastic items), are transported to shorelines by wind, waves, and ocean currents (Alam et al., 2019; Sodré et al., 2023). Plastic debris can adsorb organic and inorganic pollutants, including heavy metals, thereby acting as vectors of contamination for various organisms if ingested (Jiménez-Contreras et al., 2024; Xiang et al., 2022).

In this study, we investigate the occurrence of microplastics and mesoplastics (M(e)Ps) in the sandy beach and sediments of Praia Grande, a major tourist destination in the city of São Francisco do Sul, Santa Catarina State, Brazil. This extensive beach exhibits heterogeneous features in terms of sediment composition and seafront exposure, including both exposed and semi-sheltered shoreline areas, as well as varying levels of tourist activity. Our analysis focused on the distribution of plastic particles.

This study provides novel insights into the distribution and characteristics of M(e)Ps in southern Brazil, a region that has received limited attention with respect to this environmental issue. Our findings can contribute to a deeper understanding of plastic pollution in coastal environments and support future efforts in environmental monitoring, management, and protection.

## Materials and methods

### Study area and sampling

Samples were collected from Praia Grande, a major tourist destination located in the city of São Francisco do Sul, on the northern coast of Santa Catarina State (SC), Brazil. This beach stretches approximately 26 km along the shoreline and includes a permanent environmental preservation area associated with the Acaraí State Park.

Sampling, preparation, and characterization procedures followed protocols established by the Group of Experts on the Scientific Aspects of Marine Environmental Protection (GESAMP, 2019) and the National Oceanic and Atmospheric Administration (NOAA) (Masura et al., 2015), with modifications proposed by Besley et al. (2017) and Urban-Malinga et al. (2020).

**Fig. 1:**
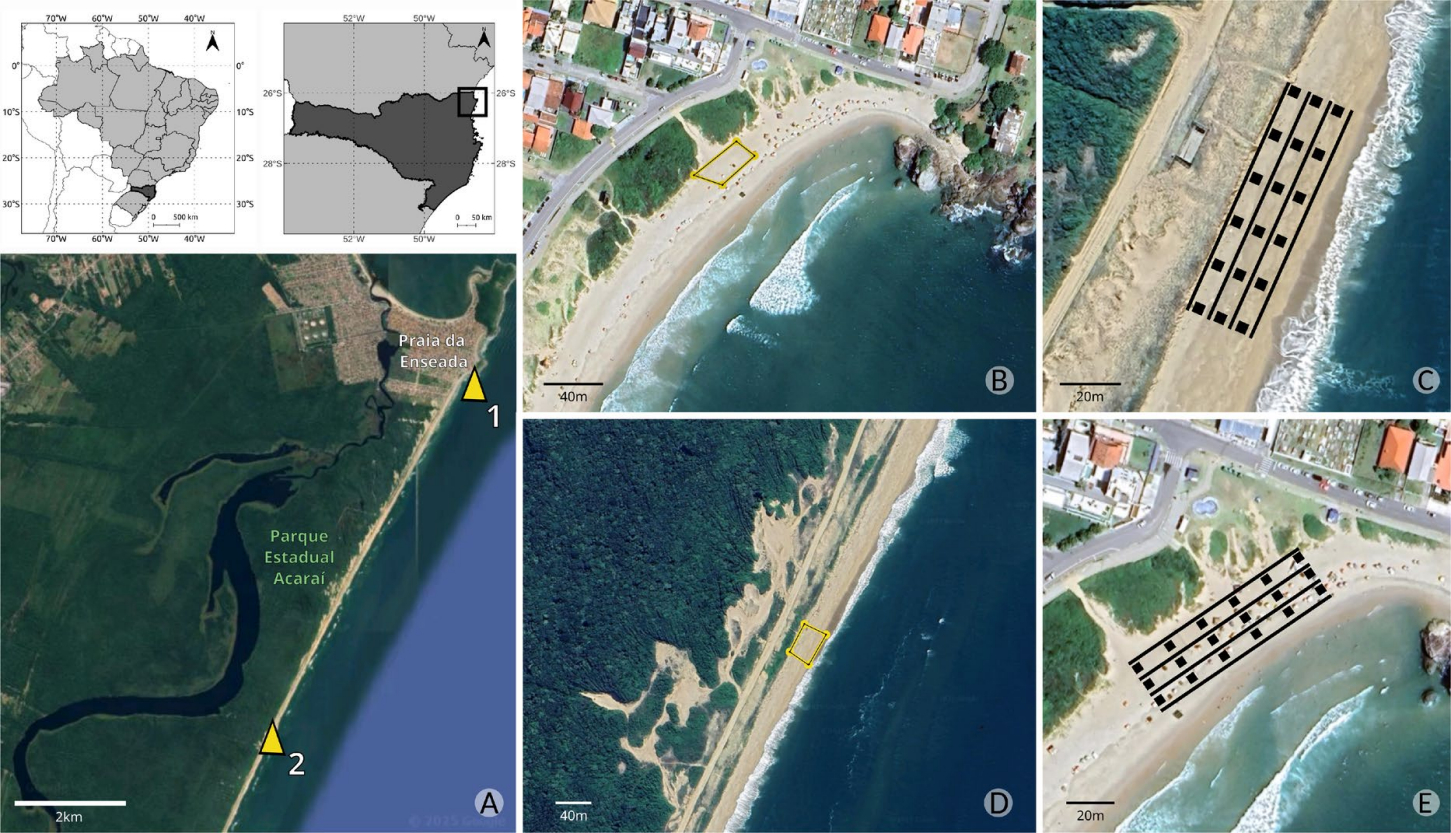
Location of beach sand sediment sampling sites in the Praia Grande region, São Francisco do Sul, Brazil, designated as Area #1 and Area #2, which are 5 km apart. **A** Overview of Praia Grande beach; **B**, **E** Close-up of Area #1 (urbanized zone); **C**, **D** Close-up of Area #2 (protected area within Acaraí State Park) Source: Adapted from Google Earth

Samples were collected along two distinct transects (Fig. 1A), each running parallel to the shoreline. The first transect, referred to as Area #1 (Fig. 1B), was located in an urbanized region and measured approximately 105 × 30 m. The second transect, Area #2 (Fig. 1D), was situated within the Acaraí State Park and measured approximately 105 × 21 m. Central sampling points for both transects were determined using a Global Navigation Satellite System (GNSS), specifically the Global Positioning System (GPS), with coordinates recorded in the WGS-84 datum. In Area #1, the coordinates were −26.2351263950, −48.5026640120, while in Area #2, they were **-**26.2915200780, −48.5367879800.

Each transect was subdivided into three zones corresponding to the low tide, intertidal, and high tide areas, following the recommendations of GESAMP (Group of Experts on the Scientific Aspects of Marine Environmental Protection). Each of these tidal zones was further subdivided vertically into three sections. From each section, two sediment samples were collected from predefined points, resulting in a total of 18 sampling points per area.

The samples were collected at a depth of up to 5 cm, using wooden frames measuring 50 cm × 50 cm, and assisted by a metal shovel. The samples were transferred to sterilized glass jars, which were then covered with aluminium foil and sealed with a metal lid before being sent for analysis. One composite sand sample of 250 g (generated from 9 collection points of each studied area) were used for granulometry and textural analyses.

To minimize the risk of sample contamination from airborne particles and handling procedures, strict quality control measures were implemented. All glassware was initially washed with detergent, rinsed three times with HPLC-grade deionized water, and oven-dried at 100 °C. During sample collection, transport, and laboratory analysis, nitrile gloves and cotton lab coats were worn at all times. Laboratory procedures were conducted within a Lutech horizontal laminar flow hood to reduce airborne particulate contamination. Work surfaces were thoroughly cleaned with 70% ethanol before each procedure.

Control samples, consisting of filtered saturated sodium chloride solution, were used to assess contamination levels in the laboratory environment. One control sample was processed for each day of laboratory analysis, and was exposed to the same conditions as the experimental samples, except that it remained uncovered with aluminum foil to assess airborne contamination specifically. Control samples were examined under a stereomicroscope, and the average number of particles detected was subtracted from the particle counts observed in the experimental samples.

### Screening – sample separation and preparation

Sample separation procedures were conducted following the protocol recommended by the NOAA (Masura et al., 2015). Sand samples were first dried in an oven at 60 °C for 48 h, then sieved using stainless steel meshes with 5.6 mm and 1 mm openings. Mesoplastics (MePs, 5–25 mm) and microplastics (MPs, 1–5 mm) were initially identified by visual inspection and manually separated using histological forceps. Sieved sand from each sampling point was homogenized and divided into 50 cm^3^ aliquots for density separation. This process was carried out using a saturated sodium chloride (NaCl) solution, prepared by dissolving 360 g of analytical-grade NaCl in 1 L of ultrapure water, then filtered through a 0.7 μm Millipore glass fiber filter. Sodium chloride is one of the most commonly used salts for microplastic density separation due to its low cost, wide availability, and minimal environmental impact (Prata et al., 2019). Filtration of the saline solution prior to use was necessary to eliminate potential microplastic contaminants, as previously demonstrated by (Yang et al., 2023) in a study on commercial table salt.

For each sample, 50 cm^3^ of dry sand was added to a 600 mL glass beaker, followed by 200 mL of the filtered saline solution, as described by Urban-Malinga et al. (2020). The mixture was stirred with a magnetic stirrer at 600 rpm for 2 min using a glass-coated stir bar, then allowed to settle for 1 h. The supernatant was carefully decanted and filtered under vacuum using glass fiber filters with a pore size of 0.7 μm. Both decantation and filtration were performed in triplicate. Filtrates were collected in covered Petri dishes and dried in an oven at 40 °C for 24 h. Throughout the stirring, sedimentation, and filtration steps, beakers and filtration funnels were covered with aluminum foil to minimize contamination.

#### Microscopic characterization and morphological classification

Microplastic (MP) particles between 1 and 5 mm and mesoplastic (MeP) particles between 5 and 25 mm were retained on sieves and initially visualized with the naked eye. These particles were quantified and measured using a digital caliper. Microscopic analysis of the filtrates was performed using an Olen stereomicroscope coupled to a digital camera (Digilab model DI-5.0HD). Image processing and particle quantification in the size range of 0.3 to 5 mm were conducted using the stereomicroscope manufacturer’s software, following NOAA (Masura et al., 2015) guidelines. Particle measurements and morphological analyses were performed at 40 × magnification.

Particle morphology (including color, shape, and size) was described according to criteria established by GESAMP (2019) and NOAA (Masura et al., 2015). Particles were classified by shape as fibers, fragments, granules/pellets, or films. Size classification was based on fiber length and the cross-sectional dimensions of other particle types. Color classification was based on the polymer pigmentation.

#### Polymer characterization via ATR-FTIR spectroscopy

Polymer characterization was conducted using attenuated total reflectance–Fourier transform infrared (ATR-FTIR) spectroscopy. A total of 105 MPs (1–5 mm) and 45 MePs were selected based on morphological similarity for polymer identification. Samples were placed directly on the ATR module using fine-tipped histological forceps. Spectral scans of the 150 samples were recorded over the wavenumber range of 650–4000 cm⁻^1^, and spectra were compared against a reference database for polymer identification. Polymer fragments were identified by their characteristic absorption of electromagnetic radiation at specific wavelengths, corresponding to functional groups unique to each polymer type. Transmittance intensity peaks were analyzed to confirm polymer composition. Analyses were performed using an Agilent Cary 630 FTIR spectrometer, referencing the manufacturer’s spectral library and literature sources (Silverstein et al., 2006; Pavia et al., 2010; Kappler et al., 2016). More specifically, the type of ATR crystal used was diamond in a wavenumber range of 4000–650 cm⁻^1^ with a spectral resolution of 8 cm⁻^1^. For each spectrum, 32 scans were performed. Background measurements were performed with the same settings. Sample identification was done using the database of the ATR-FTIR equipment library (Agilent brand, Cary 630 FTIR model). The libraries used were ATR Demo Library with 55 spectra, Agilent Elastomer O-ring and Seal Handheld ATR Library with 771 spectra, and Agilent Polymer Handheld ATR Library with 186 spectra. The software used by the manufacturer allows comparison with the samples; however, the conclusion was supplemented by a point analysis of the functional groups according to the literature involving functional groups. The FTIR-ATR relative transmittance spectrum reports of the polymers analyzed in the research showed a quality index for comparison of the analyzed spectra with precision and accuracy in the range between 70 and 100% compared to standard spectra.

#### Statistical analyses

Statistical analyses were conducted using JAMOVI software (version 2.2.5) to evaluate differences in the abundance of MPs and MePs between Areas #1 and #2. Data normality was assessed using the Shapiro–Wilk test. Given the non-normal distribution of the data, non-parametric tests were employed: the Wilcoxon-Mann–Whitney U test to compare means between the two locations, and the Kruskal–Wallis test to assess differences across tidal zones. Spearman’s rank correlation was used to analyze the relationship between MP and MeP quantities in both areas. Statistical significance was determined at a confidence level of 95% (p ≤ 0.05). A principal component analysis (PCA) (Pearson correlation) (Sigma Plot 14) was performed for correlates polymer distribution and sediment granulometry.

## Results

### Abundance of MPs and MePs

A total of 616 plastic particles, comprising both microplastics (MPs) and mesoplastics (MePs), were collected from the two distinct sampling areas. These areas differ notably in terms of urbanization, tourism activity, and sediment granulometry. The majority of the particles (approximately 81.2%) measured less than 5 mm and were classified as MPs, while 18.8% ranged from 5 to 25 mm and were classified as MePs.

**Fig. 2:**
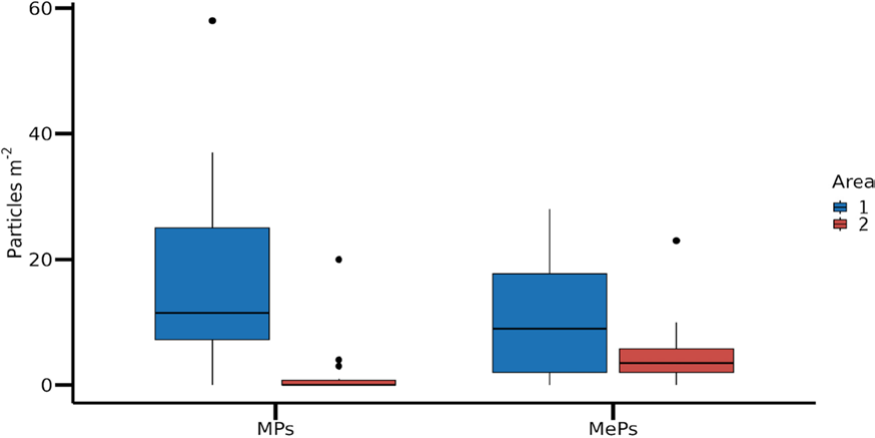
Quantity of microplastic (MP) and mesoplastic (MeP) particles collected from sand sediments at sampling sites in Areas #1 and #2 of Praia Grande beach

Significant differences were observed between Areas #1 and #2 regarding the concentration of both MPs and MePs (p ≤ 0.001) (Fig. 2). Area #1 exhibited higher concentrations of MPs and MePs compared to Area #2. Specifically, MP concentration in Area #1 was 70.44 particles·m⁻^2^ (equivalent to 7.04 particles·kg⁻^1^ of sand), while MePs were present at 25.11 particles·m⁻^2^ (2.51 particles·kg⁻^1^ sand). In contrast, Area #2 had MP concentrations of 19.11 particles·m⁻^2^ (1.91 particles·kg⁻^1^ sand) and MePs concentrations of 6.67 particles·m⁻^2^ (0.67 particles·kg⁻^1^ sand).

**Fig. 3:**
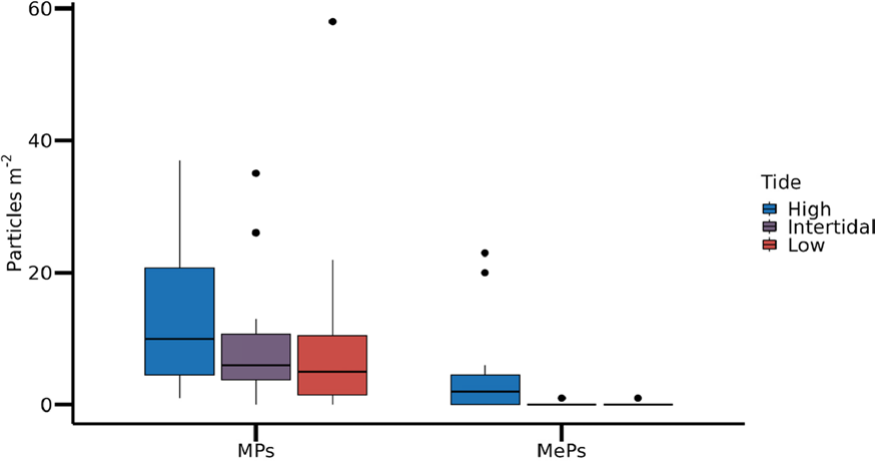
Quantity of microplastic (MPs) and mesoplastic (MePs) particles obtained in the different tidal ranges (High, Intertidal and Low) covering the two sampling areas

Regarding the different tidal ranges, significant differences were observed only for mesoplastics (MePs), as indicated by the Kruskal–Wallis test (p < 0.012), with a higher abundance of particles found in the high tide zone. In contrast, the abundance of microplastics (MPs) did not differ significantly across tidal ranges (p ≥ 0.395). The distribution of particle counts across tidal zones is shown in Fig. 3. Additionally, there was a positive correlation between the abundance of MPs and MePs in the two sampling areas (ρ = 0.623; p < 0.001).

### Shape, color, and composition of MPs and MePs

**Fig. 4:**
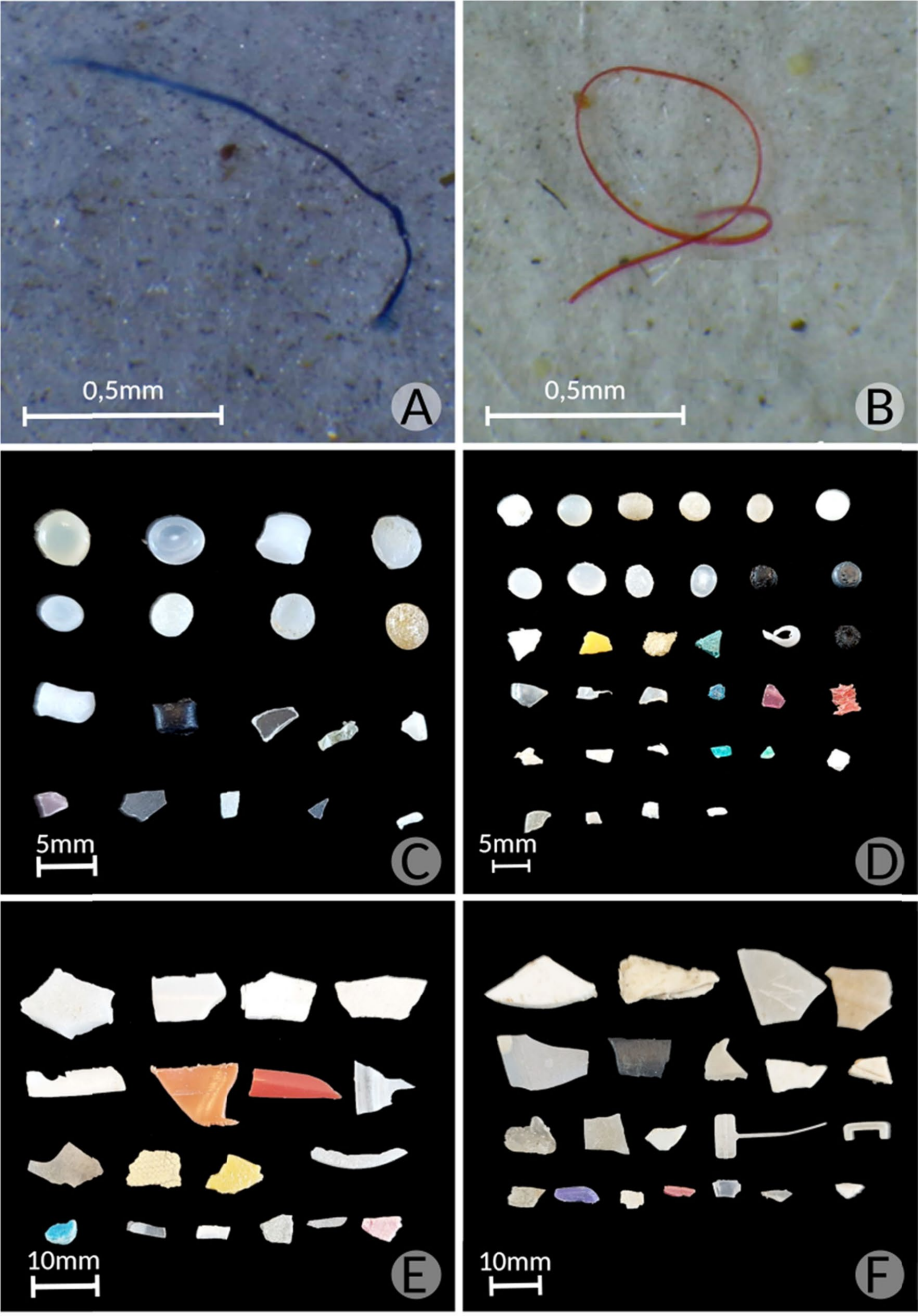
Visualization of microplastic particles under a stereomicroscope at 40 × magnification: panels (**A**) and (**B**). Different morphological aspects of microplastic particles (**C**) and (**D**), and mesoplastic particles (**E**) and (**F**), retained in the 0.5 mm sieve from sand sediment samples collected in Areas #1 and #2

The morphological characteristics of microplastics (MPs) and mesoplastics (MePs) showed considerable diversity in terms of shape, color, and size, as illustrated in Fig. 4. The samples reveal a wide variety of forms and hues, reflecting the heterogeneous nature of plastic pollution in the study area.

**Fig. 5:**
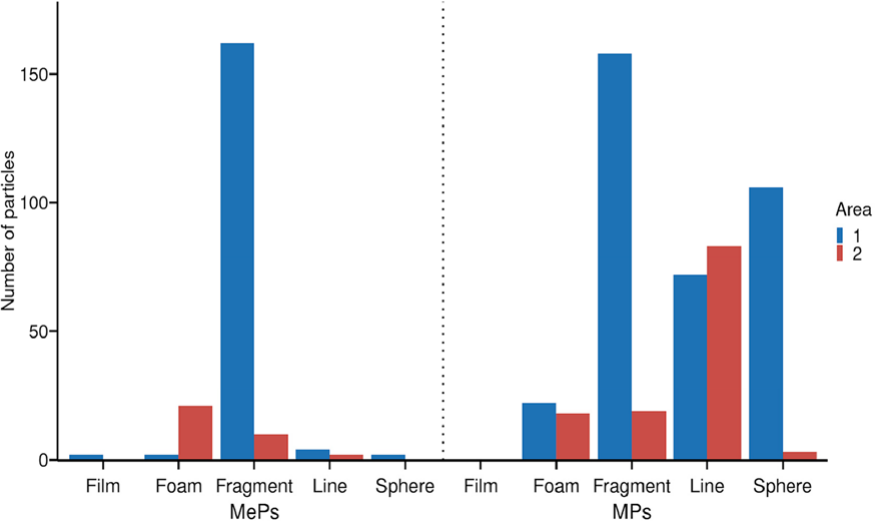
Shapes observed in microplastic (MPs) and mesoplastic (MePs) particles obtained from sand sediments collected at sampling sites in Areas #1 and #2

With respect to plastic shapes (Fig. 5), in Area #1 the fragment type was the most common, representing 49.7% of all MPs and 93.6% of MePs, followed by pellets (44.1% of MPs and 2.3% of MePs), lines (29.6% of MPs and 1.2% of MePs), foams (6.1% of MPs and 1.2% of MePs), and films, observed only in MePs, representing 1.2% of these particles.

In Area #2 (Fig. 5), the predominant shape for MPs was line type (67%), followed by fragments (15.4%), foams (14.6%), and pellets (2.4%). For MePs in Area #2, the most common shape was foam (63.6%), followed by fragments (30.3%) and lines (6.1%). Considering both areas together, the order of abundance for MPs was: fragments > lines > pellets > foams. For MePs, the order was: fragments > foams > lines > pellets and films (with pellets and films present in equal amounts).

**Fig. 6:**
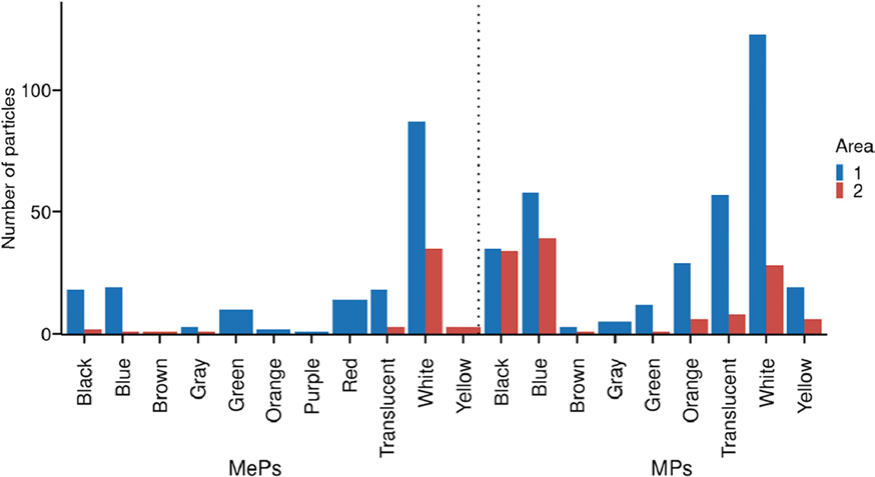
Colors observed in microplastic (MPs) and mesoplastic (MePs) particles obtained from  sand sediments collected at sampling sites in Areas #1 and #2

A total of nine plastic colors were identified in this study: white, blue, black, translucent, red, green, yellow, gray, and purple. Among the MPs in Area #1, white was the most abundant color for fragments, spheres, and foams, while blue predominated in line-shaped particles. In Area #2, white was the most common color for fragment and sphere-shaped MPs, whereas blue was predominant for line-shaped MPs. In both areas, 100% of foam-type MPs were classified as white (Fig. 6).

Regarding MePs, white was the most abundant color for fragments, lines, films, and foams in Area #1. For sphere-shaped MePs in Area #1, blue and white were present in equal proportions, and again, 100% of foam-type MePs were white. In Area #2, white was the predominant color for fragment- and foam-type MePs, while line-shaped MePs were mostly translucent and white, both occurring in equal proportions.

**Fig. 7:**
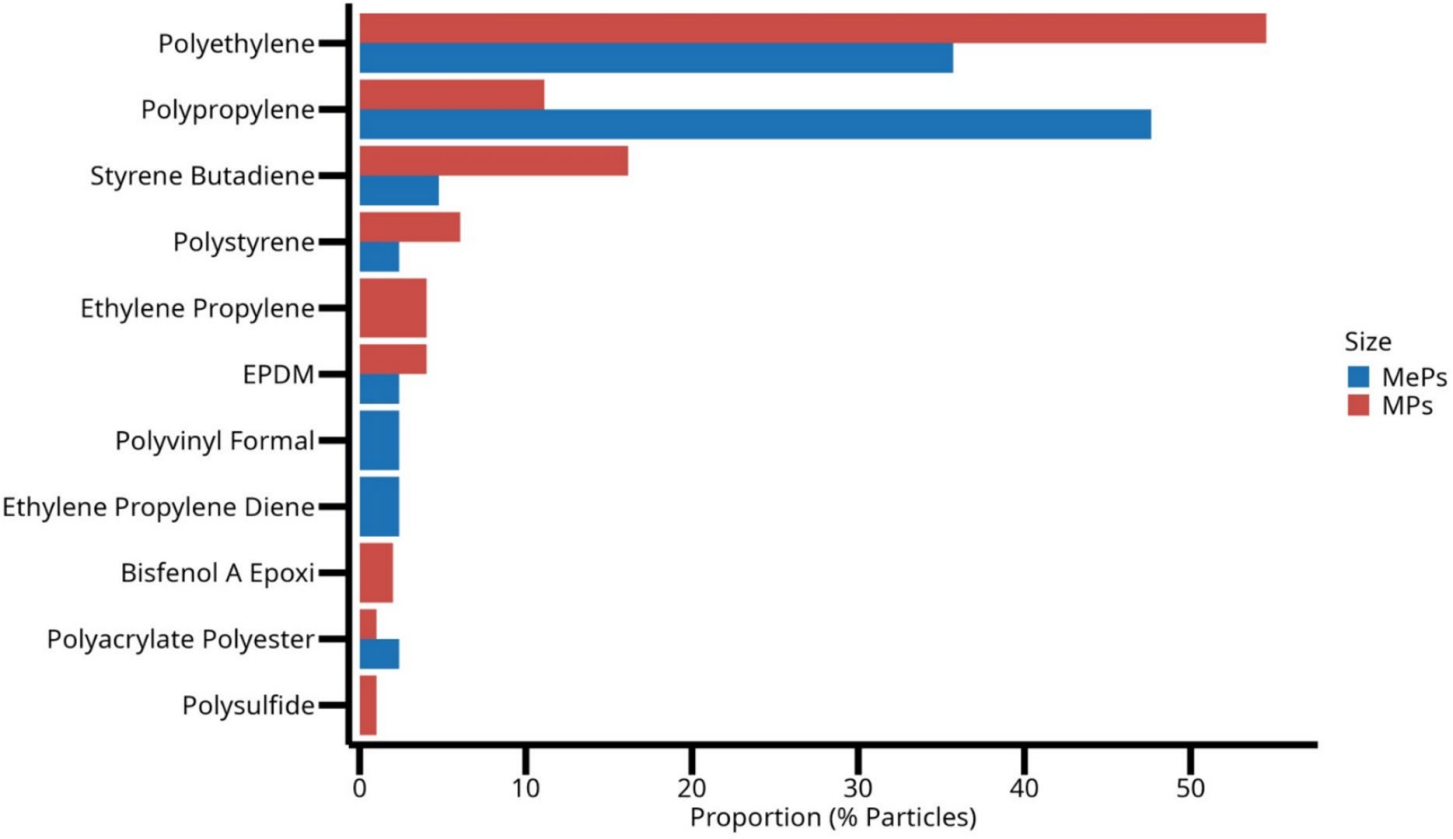
Microplastic (MPs) and mesoplastic (MePs) polymers proportions in the beach sand sediments of Areas #1 and #2 identified with ATR-ATR spectroscopy

Fourteen different types of polymers were identified in the samples analyzed via ATR-FTIR. The most abundant M(e)P polymers were polyethylene (51.4%), styrene-butadiene copolymer (16.2%), and polypropylene (15.2%) (Fig. 7). For MePs specifically, polypropylene was the most abundant polymer (46.5%), followed by polyethylene (34.9%) and styrene-butadiene copolymer (4.6%), with other polymer types present in smaller quantities. Together, polyethylene, polypropylene, and styrene-butadiene copolymer accounted for 80.27% of all polymers identified in this study.

**Fig. 8:**
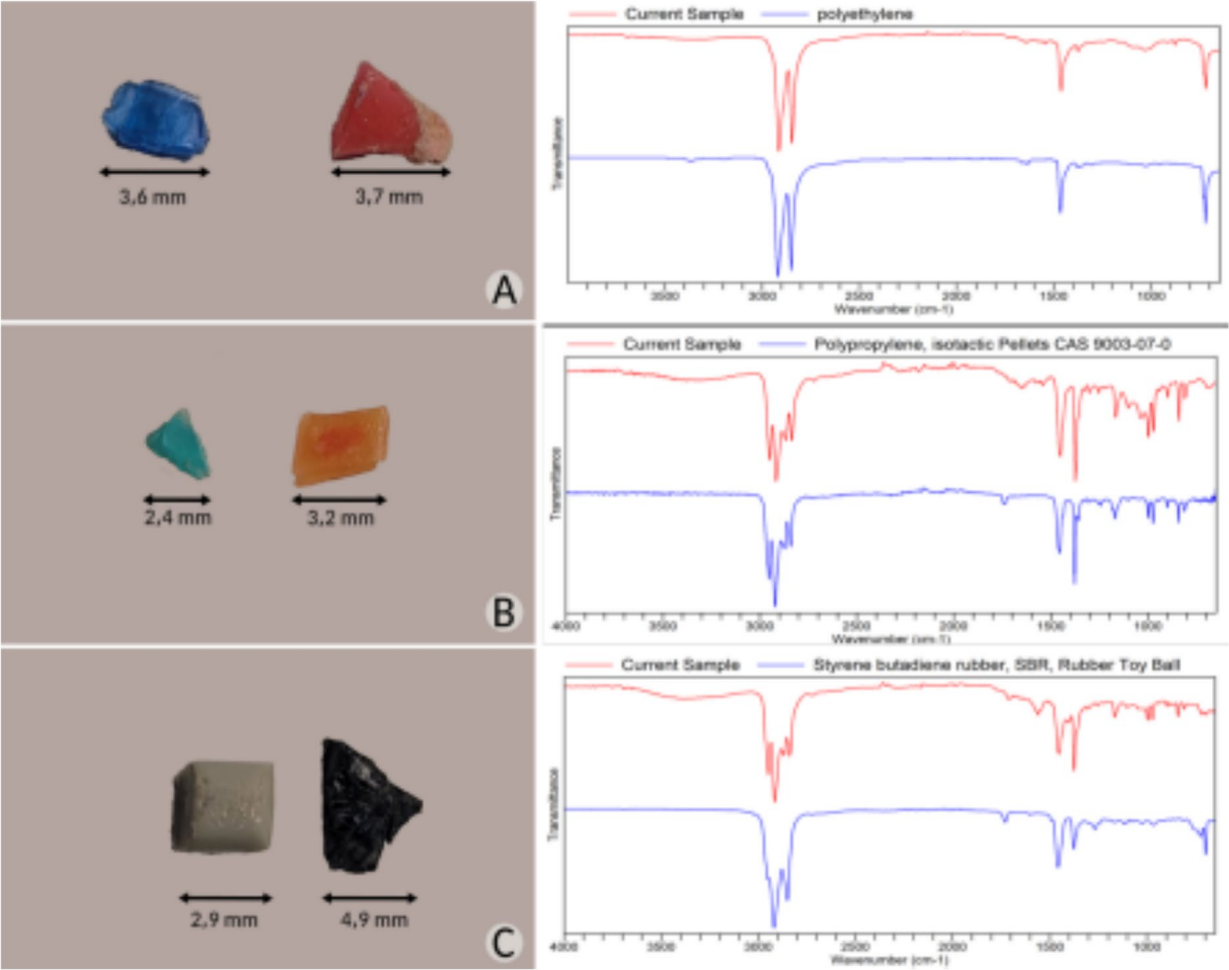
Selected ATR-FTIR transmittance spectra of main polymers detected in the spectral range of 650 to 4000 cm^-1^. Panel **A**: Polyethylene; Panel **B**: Polypropylene; Panel **C**: Styrene butadiene rubber

The relative ATR-FTIR transmittance spectra of the main polymers detected in this study are shown in Fig. 8. In the supplementary material section are showed in Fig. 8S the mean identified polymers, along with their corresponding HQI values derived from spectral recognition.

### Granulometry of sand sediments

**Fig. 9:**
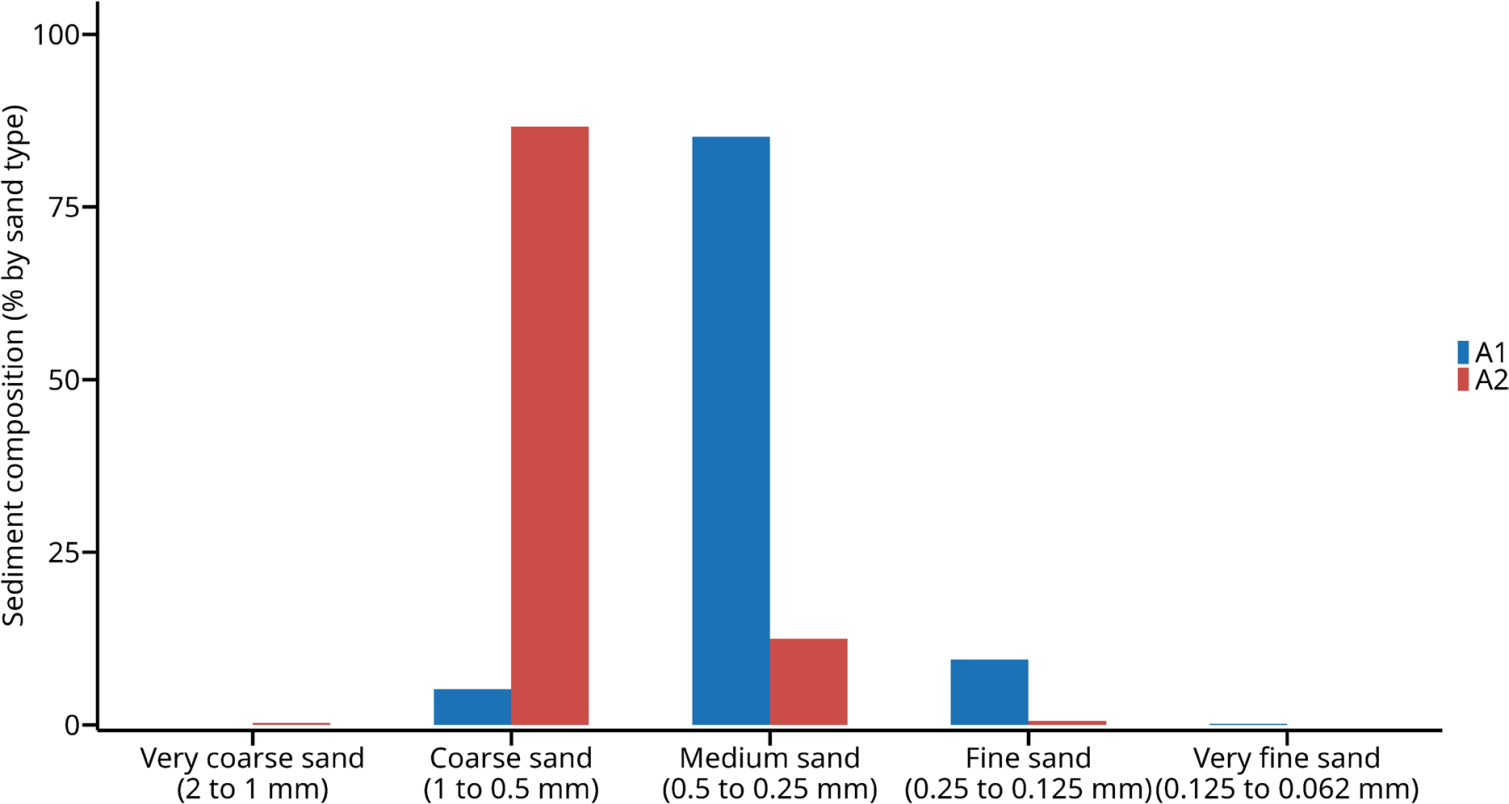
Composition of beach sand sediments collected from sampling sites in Area #1 (A1) and Area #2 (A2) based on granulometry analysis

The composite sand samples from the two studied areas showed significant differences in relation to granulometry (Fig. 9). Samples from Area #1 had a greater abundance of medium sand (0.5 to 0.25 mm) (85.2%), followed by fine sand (0.25 to 0.125 mm) (9.5%), coarse sand (1 to 0.5 mm) (5.2%), and very fine sand (0.125 to 0.062 mm) (0.2%). Area #2 had a greater abundance of coarse sand (1 to 0.5 mm) (86.6%), followed by medium sand (0.5 to 0.25 mm) (12.5%), fine sand (0.25 to 0.125 mm) (0.6%), and very coarse sand (2 to 1 mm) (0.3%).

## Discussion

Before discussion of the results of this study, it is worth remembering that, in any experimental work, there are some limitations in the sampling and characterization of microplastics and mesoplastics (Hidalgo-Ruz et al., 2012; Mecozzi et al., 2016; Shim et al., 2017). Thus, in relation to the potential sampling biases, this action is often restricted to specific sites or times, which may not represent the heterogeneity of MPs/MePs distribution. Furthermore, nets with different mesh sizes (e.g., 333 µm vs. 100 µm) capture different fractions of particles. This leads to a bias toward larger particles when coarser meshes are used. Also, fibers from clothing or equipment can introduce artificial MPs, especially in low-concentration environments, inflating particle counts. Concerning to minimum detectable particle size, visual sorting is typically limited to particles > 300–500 µm, whereas spectroscopic techniques like FTIR or Raman can detect particles down to ~ 10–20 µm, depending on the equipment and sample preparation. As a result, studies may systematically underestimate the abundance of smaller MPs, which can be ecologically significant due to their higher bioavailability and mobility. Regarding polymer identification, many studies rely on visual identification, which can misclassify natural fibers, degraded plastics, or other particulate matter as MPs/MePs. Spectroscopic instrumental methods (FTIR, Raman) can accurately identify polymer types but are time-consuming and may require subsampling, introducing representativeness issues. Weathered or biofouled plastics can produce spectra that are difficult to match with reference libraries, leading to uncertainties in polymer composition. There are also uncertainties related to MP/MeP separation. In this sense, density separation, often used to isolate plastics from sediments or organic matter, may fail for plastics with densities near that of the separation medium (e.g., PVC, PET), and agglomeration of particles, adherence to organic debris, or fragmentation during extraction can lead to underestimation of particle counts or misclassification of sizes. Thus, distinction between mesoplastics and microplastics is sometimes arbitrary, as size thresholds (e.g., 1–5 mm for MePs, < 1 mm for MPs) vary between studies, complicating comparisons. Quantification and representativeness of debris can be problematic due to the subsampling of extracted particles for chemical analysis, and reported concentrations often reflect estimates rather than exact counts. Finally, heterogeneity in environmental matrices (water column, sediments, biota) adds high variability, limiting the reproducibility and comparability of results across studies.

### Anthropogenic pressures as local modulators

The two study areas differ markedly in terms of urbanization intensity, tourism pressure, and proximity to port and fishing activities, providing a relevant context to evaluate the influence of anthropogenic drivers on plastic accumulation. Area #1, characterized by greater urban occupation and recreational use, exhibited significantly higher concentrations of both microplastics (MPs) and mesoplastics (MePs) compared to the less urbanized Area #2. This pattern suggests that local human presence acts as an important modulating factor in the input and accumulation of plastic debris.

Urbanized coastal zones are widely recognized as focal points of plastic release due to inadequate waste disposal, recreational activities, stormwater runoff, and port-related operations. Studies conducted in Brazilian beaches, such as those in Niterói (RJ State) (Castro et al., 2020), have reported elevated plastic densities in areas with intense human activity, reinforcing this relationship. Additionally, artisanal and recreational fishing activities have been identified as relevant contributors to coastal plastic inputs, particularly in areas adjacent to fishery zones (Dowarah & Devipriya, 2019; Krüger, et al., 2020). In this context, the predominance of fibers in Area #2 may reflect fishing-related sources rather than urban discharge, indicating that different anthropogenic activities generate distinct morphotype signatures.

Morphological patterns further support the influence of local human activities. In Area #1, fragments were the dominant morphotype among both MPs and MePs. High proportions of fragments are typically associated with secondary plastics derived from the degradation of larger debris, frequently linked to tourism and urban littering (Key et al., 2024; Turra et al., 2014). The notable presence of pellet-type MPs in Area #1 may also be influenced by the proximity of the Port of São Francisco do Sul and the Port of Itapoá, where industrial resin handling and maritime transport increase the risk of accidental pellet loss. In contrast, the predominance of line-type MPs and foam-type MePs in Area #2 suggests inputs associated with fishing gear and buoyant packaging materials.

Despite these patterns, it is important to recognize that urbanization alone does not fully explain plastic accumulation. Some authors have reported that microplastic presence in beach sediments may occur independently of direct urban influence (Andrady, 2011), while others emphasize the role of estuarine proximity and hydrodynamic transport as key determinants of debris deposition (Andrades et al., 2020). Therefore, although anthropogenic pressure clearly influences plastic abundance and morphotype composition in the studied transects, its effect appears to be context-dependent and mediated by local environmental conditions.

Overall, the comparison between Areas #1 and #2 indicates that human activities act as a significant but secondary control on plastic distribution, shaping input typology and particle characteristics, while interacting with broader physical processes that regulate retention and spatial sorting.

### Physical controls as first-order drivers

Sediment granulometry and hydrodynamic conditions emerged as the primary controls on the spatial distribution of both microplastics (MPs) and mesoplastics (MePs) in Praia Grande. Area #1 was dominated by medium sand (0.25–0.5 mm), whereas Area #2 presented a higher proportion of coarse sand (0.5–1 mm). These differences in sediment texture play a crucial role in particle retention, as finer sediments provide greater surface area and lower hydrodynamic energy, favoring the accumulation of lightweight and highly fragmentable polymers such as polyethylene and polypropylene (Alomar et al., 2016; Molazadeh et al., 2024; Rodrigues et al., 2024).

**Fig. 10:**
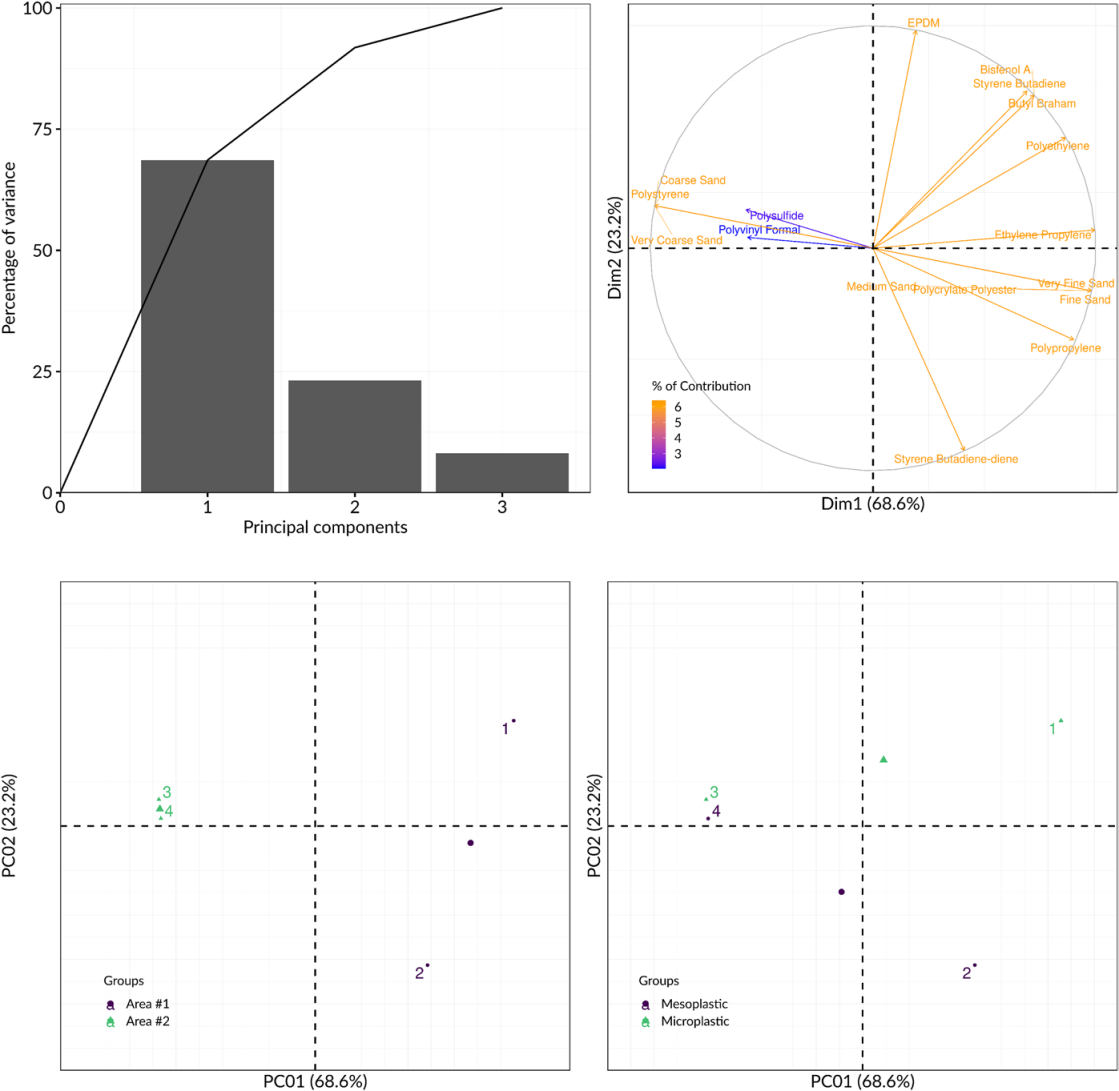
Principal component analysis in relation to the polymer distribution and sediment granulometry

Hydrodynamic factors, including seafront exposure and tidal range, further modulate plastic deposition. MPs and MePs were found at higher concentrations in the high-tide zones compared to intertidal and low-tide areas. Semi-sheltered conditions in Area #1 promote particle retention, whereas the more exposed Area #2 favors dispersal and reduced deposition (Godoy et al., 2020). These observations indicate that local beach morphology and exposure patterns strongly interact with sediment characteristics to determine where plastics accumulate. The distribution range of MP concentrations in beach sediments verified in this study is similar to other studies carried out in Latin America, according to the survey by Fernandes et al. (2022), which showed variations between 0.000029 and 88.224 particles.m^−2^ of sediment (or 0.004 to 660 particles.kg^−1^ of dry sediment). Due to the lack of standardization involving sampling methodologies and the use of different units of measurement, it is complex to carry out a systematic comparison with previous studies (Carvalho, et al., 2021; Montagner et al., 2021; Oliveira et al., 2023). A principal component analysis in relation to the polymer distribution and sediment granulometry is shown in Fig. 10.

Principal Component Analysis (PCA) reinforced this hierarchy of controls. PC1, explaining 68.6% of the variance, corresponded to sediment granulometry and associated physical sorting mechanisms. Fine and very fine sands were strongly correlated with polyethylene and polypropylene fragments, consistent with the preferential deposition of low-density particles in low-energy environments. Coarser sands were associated with stiffer or heavier polymers such as polystyrene and polysulfide, indicating selective retention under higher-energy conditions (Fenn et al., 2025; Uddin et al., 2019). PC2 (23.2%) captured variation in polymer type related to source characteristics, distinguishing industrial or elastomeric materials (e.g., EPDM, styrene-butadiene) from packaging-derived polymers. However, sediment granulometry and hydrodynamics clearly exerted the dominant influence, demonstrating that physical conditions constitute the first-order driver of plastic accumulation.

In summary, these findings highlight that while anthropogenic activities provide input, it is the physical environment—sediment texture, wave action, beach exposure, and tidal influence—that primarily determines plastic retention and spatial distribution. This distinction reinforces the need to consider local sedimentary context in coastal monitoring and predictive assessments of plastic pollution.

### Source typology and polymer signature

The analysis of polymer types revealed distinct patterns associated with the source and use of plastics in the two study areas. Fourteen polymer types were identified using ATR-FTIR, with polyethylene (PE), polypropylene (PP), and styrene-butadiene copolymer (SBR) collectively representing more than 80% of all particles. Area #1 was dominated by PE, followed by SBR and PP, while Area #2 exhibited a predominance of PP, followed by PE and SBR. These distributions reflect both local inputs and global production trends, as PE and PP account for nearly half of total worldwide plastic production (Plastics Europe, 2023).

The PCA results suggest that PC2 (23.2% of variance) captures differences related to polymer origin rather than sedimentary control (Fig. 10). Positive loadings were associated with elastomeric and industrial-related materials (e.g., SBR, EPDM), commonly linked to urban infrastructure, vehicular wear, and industrial activities. Negative loadings were dominated by ubiquitous packaging-derived polymers such as PE and PP. This axis indicates that anthropogenic sources impart a recognizable signature to polymer assemblages, distinguishing industrial and urban inputs from widely dispersed consumer plastics.

Morphological and color characteristics further support source differentiation. Area #1, with higher urbanization and port proximity, exhibited a predominance of fragments and pellets, reflecting secondary degradation of packaging and industrial pellets. In contrast, Area #2 showed greater presence of line-type MPs and foam MePs, consistent with fishing-related debris and buoyant packaging materials. Color analysis indicated that white, blue, and translucent particles were most abundant, consistent with widespread packaging materials and fishing gear, while dark and pigmented plastics were less common. Colored microplastics are considered a risk to the health of the biota, since they can be mistaken as food by many animal species (Abidli et al., 2018; Bessa et al., 2018; Ugwu et al., 2021). Different species of animals can be more or less vulnerable depending on the color of the polymer. For example, it has been shown that white, clear, and blue microplastics are the main ones ingested by herbivorous fish (Boerger et al., 2010; Browne et al., 2008). Seabirds tend to eat light-colored microplastics due to similarities with their foods, such as fish eggs and shellfish, while white microplastics with a translucent appearance are commonly ingested by oysters (Digka et al., 2018; Li et al., 2018).

The polymeric composition has important implications for environmental persistence and ecological risk. PE and PP are low-density and highly fragmentable, facilitating transport and incorporation into sediments as secondary microplastics. SBR and other elastomeric polymers are more resistant to fragmentation but may carry associated chemical additives or adsorbed pollutants. Therefore, the polymer signature not only reflects source typology but also informs predictions regarding degradation dynamics, secondary MP formation, and potential toxicological exposure to coastal biota (Akarsu, 2023; Leistenschneider et al., 2023).

Overall, these findings indicate that while sedimentary and hydrodynamic conditions govern particle accumulation (4.2), the polymer type and source characteristics provide a complementary layer of information that identifies dominant anthropogenic contributions and potential environmental risks, bridging the understanding between local inputs and observed spatial patterns.

### Mesoplastics as transitional reservoirs

Mesoplastics (MePs) play a crucial role in the coastal plastic continuum, acting both as contemporary pollutants and as precursors of future secondary microplastics (MPs). In Praia Grande, MePs represented 18.8% of total plastic particles, with their abundance positively correlated with MPs across both study areas (ρ = 0.623; p < 0.001). This correlation indicates that MePs not only co-occur with MPs but may contribute dynamically to the generation of secondary microplastics through environmental degradation processes, including ultraviolet radiation, mechanical abrasion, and hydrodynamic stress (Song et al., 2017).

Spatial patterns further illustrate the transitional role of MePs. In Area #1, urbanized and semi-sheltered, MePs were predominantly fragments and showed higher retention in high-tide zones, reflecting both anthropogenic input and the physical sorting imposed by beach morphology. In Area #2, MePs were largely foam types associated with fishing and recreational activities, highlighting that source type influences both the physical form and the deposition dynamics of mesoplastics. The variation in shape and density affects sediment integration and the potential for gradual breakdown into smaller particles, reinforcing MePs as reservoirs of future MPs.

Comparative studies reinforce the ecological and environmental relevance of MePs. Beaches in northeastern Tunisia and the Massaciuccoli Natural Park (Italy) reported MeP densities of 36.26 ± 49.67 particles·m⁻^2^ and 100 ± 44 particles·kg⁻^1^, respectively, both higher than those observed in Praia Grande (40.7 particles·m⁻^2^ or 4.07 particles·kg⁻^1^) (Abdelkader et al., 2023; Scopetani et al., 2021). Observations from the Korean Peninsula and the Peruvian coast also demonstrate consistent positive associations between MPs and MePs (De-la-Torre et al., 2023; Lee et al., 2015), emphasizing the generality of this transitional relationship in coastal environments.

The role of MePs as transitional reservoirs has important implications for environmental monitoring and pollution management. Areas with high MeP accumulation are likely to be hotspots for future secondary microplastic formation, representing both an immediate ecological concern and a predictor of long-term contamination trends. Integrating MeP distribution into monitoring frameworks enables a process-based assessment, linking present-day plastic load to potential future microplastic generation, and informing targeted management interventions to mitigate environmental impacts.

In summary, the presence and characteristics of mesoplastics in Praia Grande highlight their dual role as both pollutants and progenitors of secondary microplastics. By acting as transitional reservoirs, MePs bridge the gap between primary debris inputs and the finer-scale microplastic pollution, underscoring the need to consider their dynamics in coastal plastic assessments and management strategies.

### Integrated conceptual framework and comparative synthesis

The synthesis of our findings highlights a hierarchical interaction between physical and anthropogenic drivers shaping the distribution and composition of micro- and mesoplastics in Praia Grande. Sediment granulometry and hydrodynamic conditions emerge as first-order controls, determining the retention, sorting, and polymer-specific accumulation of plastic debris. Fine- to medium-grained sands in Area #1 favor the deposition of low-density, fragmentable polymers such as polyethylene and polypropylene, whereas coarser sediments in Area #2 are associated with heavier or more rigid polymers. Hydrodynamic exposure and tidal influence further modulate these patterns, with sheltered and high-tide zones promoting accumulation while exposed areas enhance dispersal.

Anthropogenic pressures act as secondary modulators, influencing the typology, abundance, and morphotype of plastics rather than their primary spatial distribution. Urbanization, tourism, and port activities in Area #1 generate a higher prevalence of fragments and pellets, reflecting both secondary degradation and industrial inputs. In contrast, Area #2, with limited urban activity, shows a dominance of fibers and foam-type MePs, consistent with fishing-related debris. These patterns demonstrate that local human activity leaves a recognizable signature on particle morphology and source typology, but its effect is mediated by the prevailing physical environment.

Mesoplastics integrate these dynamics as transitional reservoirs, linking present-day anthropogenic inputs with future microplastic generation. The positive correlation between MePs and MPs suggests that areas with higher MeP abundance serve as sources of secondary MPs, emphasizing the dynamic and temporally extended nature of coastal plastic contamination. International comparisons corroborate this pattern, with similar MeP-MP relationships observed in diverse coastal settings, indicating the generality of this phenomenon.

By conceptualizing the interactions between physical controls, anthropogenic inputs, and MeP dynamics, a process-based framework emerges:

Physical controls (sediment granulometry, hydrodynamics, tidal exposure) > Anthropogenic modulation (urbanization, tourism, ports, fishing) > MePs as transitional reservoirs → resulting in observed MPs distribution.

This framework provides a clear hierarchical understanding of plastic accumulation in Praia Grande, demonstrating that effective monitoring and management strategies must integrate both sedimentary context and local human pressures. Adopting this integrated approach enables identification of hotspot areas, prediction of secondary microplastic formation, and prioritization of mitigation measures, supporting science-based coastal management and pollution reduction initiatives.

## Conclusions

This study demonstrates that the distribution and composition of microplastics (MPs) and mesoplastics (MePs) in Praia Grande are governed by a hierarchical interaction between physical and anthropogenic factors. Thus, in relation to physical controls as primary drivers, sediment granulometry and hydrodynamics exert first-order influence on plastic retention. Medium sands and semi-sheltered conditions in Area #1 promoted higher accumulation of low-density and fragmentable polymers, whereas coarser sands and higher exposure in Area #2 limited retention. High-tide zones showed consistently greater plastic deposition. Anthropogenic pressures are modulators, thus urbanization, tourism, and port activities contributed to elevated concentrations of fragments and pellets in Area #1, while Area #2 displayed a predominance of fibers and foam MePs, reflecting fishing-related debris. Local human activities shape particle typology and source signatures but are secondary to physical environmental controls. Mesoplastics are transitional reservoirs, since MePs positively correlated with MPs in both areas, highlighting their role as precursors of secondary microplastics. Their morphology and polymer composition reflect both source input and susceptibility to environmental degradation, reinforcing the need to include MePs in monitoring programs. Thus, integrating physical conditions, anthropogenic inputs, and MeP dynamics provides a predictive, process-based framework. This approach enables the identification of accumulation hotspots, guides mitigation strategies, and informs science-based coastal management, including waste management, environmental education, and legislative interventions. In conclusion, the hierarchical interplay between sedimentary context, hydrodynamic processes, and localized human activity governs plastic accumulation in Praia Grande. Recognizing these relationships allows for targeted monitoring, effective prediction of secondary microplastic formation, and the development of strategic interventions to mitigate environmental impacts in coastal areas.

## Supplementary Information

Below is the link to the electronic supplementary material.

ESM 1 (DOCX 810 KB)

## Data Availability

Data sets generated during the current study are available within the paper, its supplementary information file, and/or from the corresponding author on reasonable request.

## Declarations

All the authors have read and understood, and have complied as applicable with the statement on ethical responsibilities of authors as found in the Instructions for Authors.
