# Supplementary material for: Presence of micro-mesoplastics in beaches and sediments of São Francisco do Sul (Brazil)
Source: Environ Monit Assess. 2026 Apr 24;198(5):499. doi: 10.1007/s10661-026-15277-2 (PMC13109131; doi:10.1007/s10661-026-15277-2)
Supplement: Supplementary file 1 — (DOCX 810 KB) [file 10661_2026_15277_MOESM1_ESM.docx]

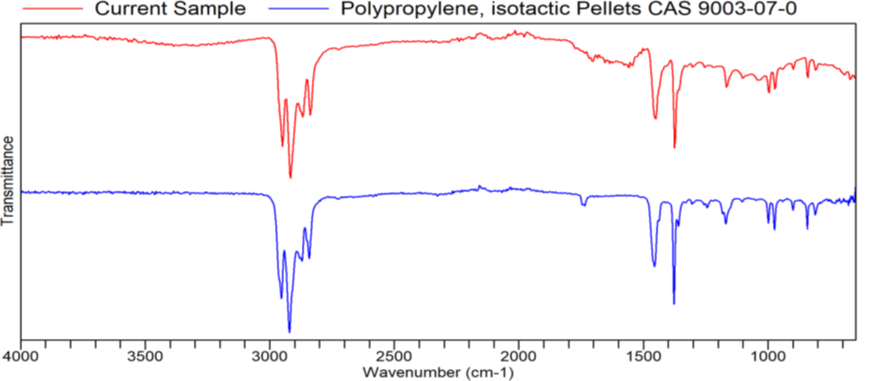


1. ATR-FTIR spectre of Polypropylene. HQI : 0.88772


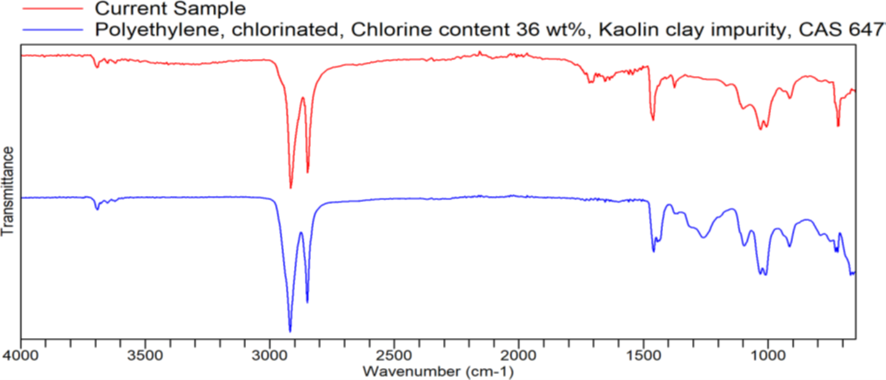


1. ATR-FTIR spectre of Polyethylene. HQI: 0.80886


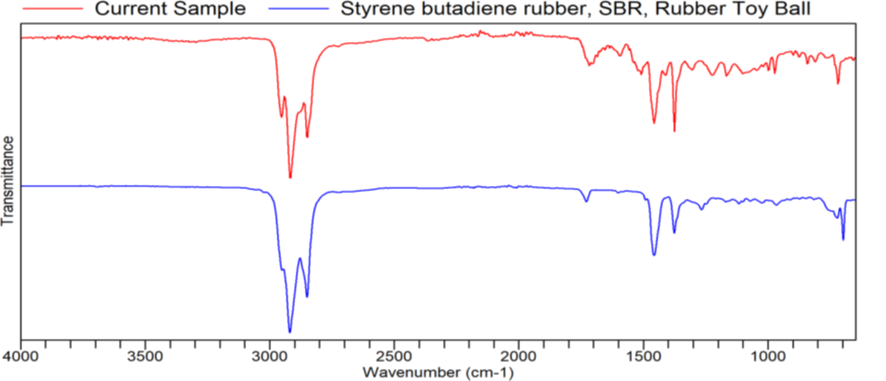


1. ATR-FTIR spectre of Styrene butadiene. HQI: 0.85149

**
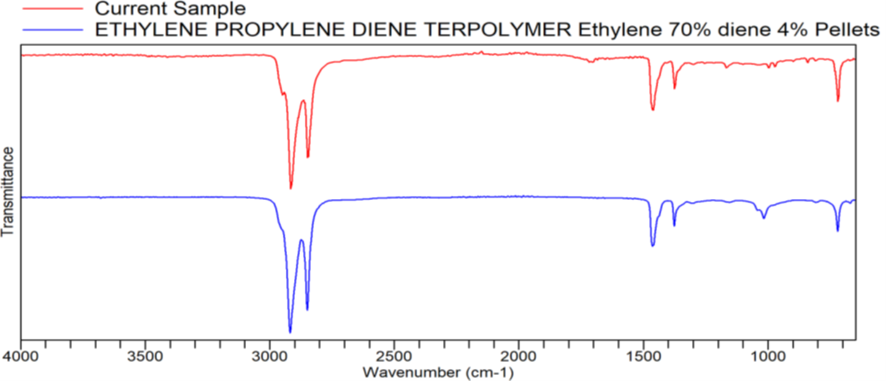
**

1. ATR-FTIR spectre of Ethylene propylene diene. HQI: 0.93234


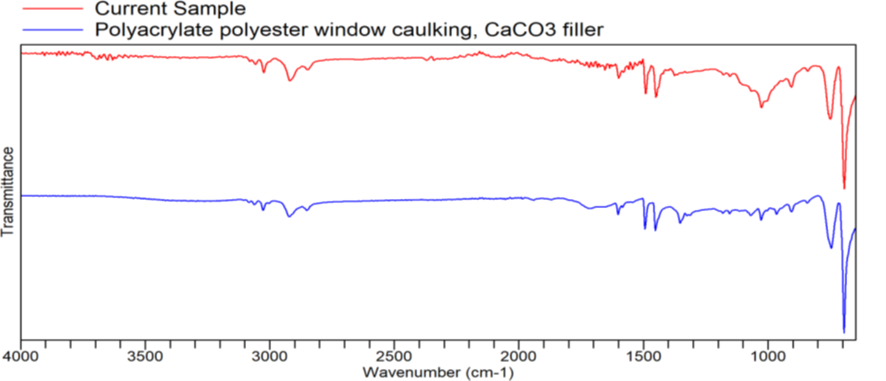


1. ATR-FTIR spectre of Polyacrylate polyester. HQI: 0.85177


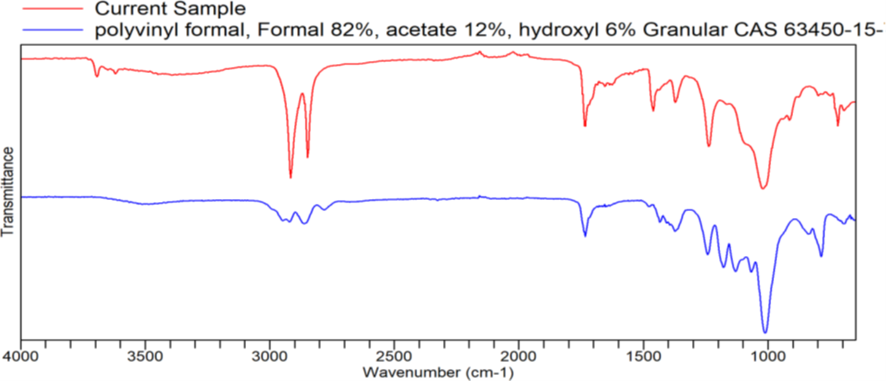


1. ATR-FTIR spectre of Polyvinyl formal. HQI: 0.75197


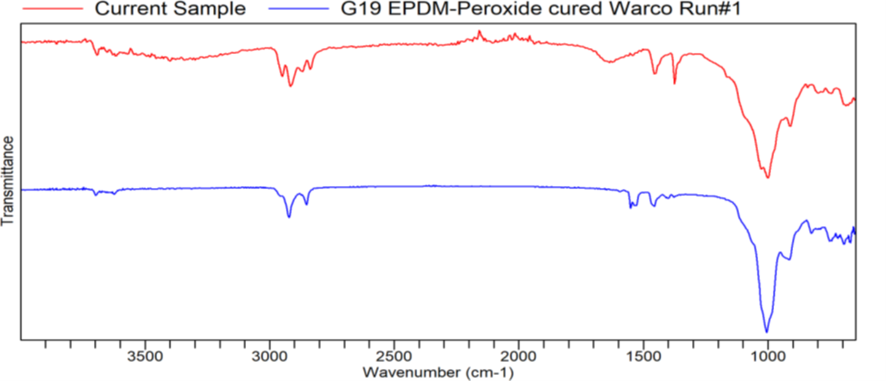


1. ATR-FTIR spectre of G19 EPDM-peroxide. HQI: 0.90299

**
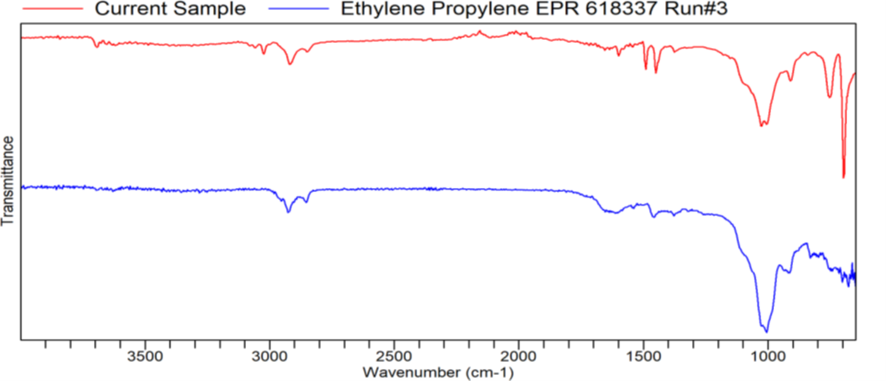
**

1. ATR-FTIR spectre of Ethylene propylene EPR 618337. HQI: 0.77643

**
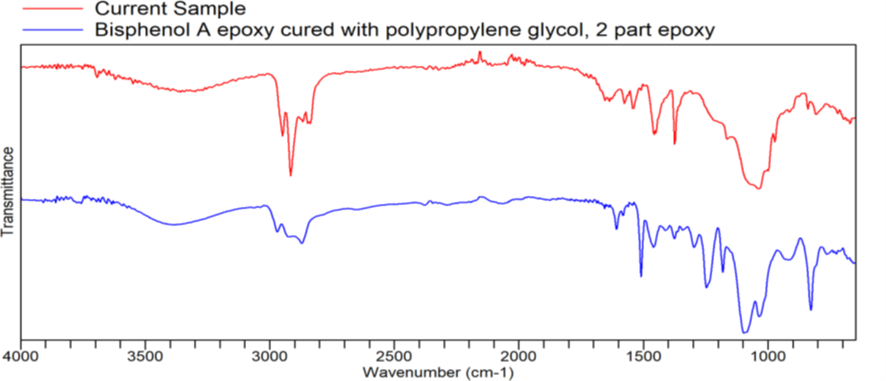
**

1. ATR-FTIR spectre of Bisphenol A epoxy. HQI: 0.77975


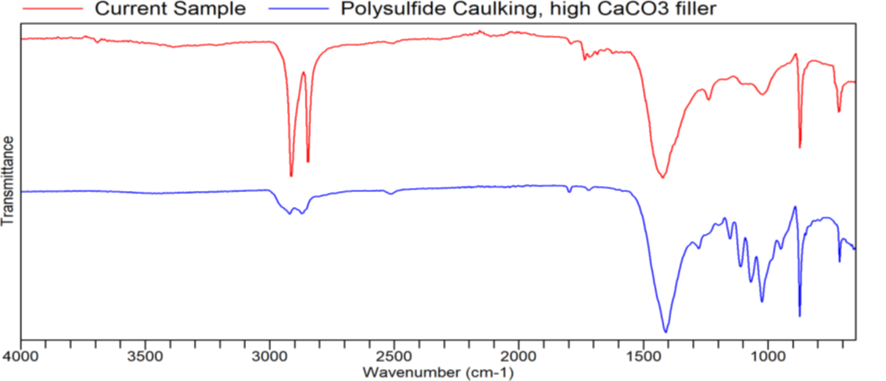


1. ATR-FTIR spectre of Polysulfide Caulking. HQI : 0.72931


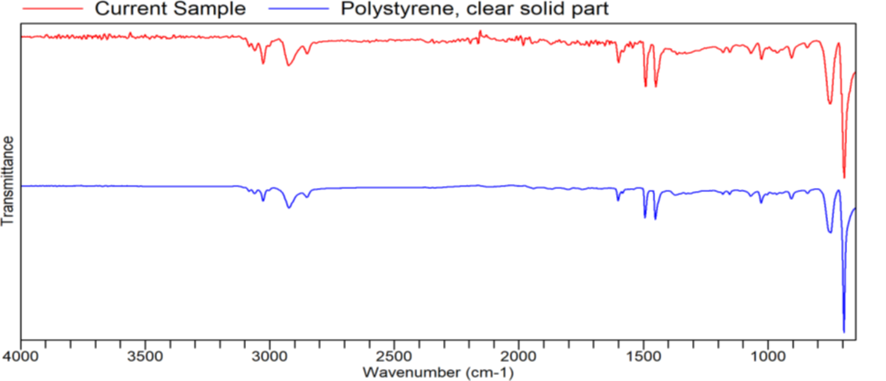


1. ATR-FTIR spectre of Polystyrene. HQI: 0.92845

**Fig. 8S** Selected ATR-FTIR transmittance spectra of main polymers detected in the spectral range of 650 to 4000 cm
